# Ribosomal protein L8 regulates the expression and splicing pattern of genes associated with cancer-related pathways

**DOI:** 10.55730/1300-0152.2666

**Published:** 2023-07-25

**Authors:** Leilei XU, Gui YANG, Bin SONG, Dong CHEN, Akbar YUNUS, Jiangtao CHEN, Xiaogang YANG, Zheng TIAN

**Affiliations:** 1 Department of Bone Tumor, The First Affiliated Hospital of Xinjiang Medical University, Urumqi, Xinjiang, P.R. China; 2 ABLife BioBigData Institute, Wuhan, Hubei, P.R. China

**Keywords:** RPL8, cancer, DEGs, alternative splicing, transcription factors

## Abstract

**Background/aim:**

Ribosomal proteins have been shown to perform unique extraribosomal functions in cell apoptosis and other biological processes. Ribosomal protein L8 (RPL8) not only has important nonribosomal regulatory functions but also participates in the oncogenesis and development of tumors. However, the specific biological functions and pathways involved in this process are still unknown.

**Materials and methods:**

RPL8 was overexpressed (RPL8-OE) in HeLa cells. MTT assay and flow cytometry were used to detect cell proliferation and apoptosis, respectively. Transcriptome sequencing was performed to analyze the differentially expressed genes (DEGs) and regulated alternative splicing events (RASEs) by RPL8-OE, both of which were validated by quantitative reverse transcription polymerase chain reaction (RT-qPCR) assay.

**Results:**

RPL8-OE inhibited cell proliferation and promoted cell apoptosis. RPL8 regulated the differential expression of many oncogenic genes and the occurrence of RASEs. Many DEGs and RASE genes (RASGs) were enriched in tumorigenesis and tumor progression-related pathways, including angiogenesis, inflammation, and regulation of cell proliferation. RPL8 could regulate the RASGs enriched in the negative regulation of apoptosis, consistent with its proapoptosis function. Furthermore, RPL8 may influence cancer-related DEGs by modulating the alternative splicing of transcription factors.

**Conclusion:**

RPL8 might affect the phenotypes of cancer cells by altering the transcriptome profiles, including gene expression and splicing, which provides novel insights into the biological functions of RPL8 in tumor development.

## 1. Introduction

Ribosomal proteins (RPs) are critical components of ribosomes. They play an important role in translation and could be combined with the unexpressed regions of mRNAs. Therefore, RPs are more involved in regulating specific transcription and translation processes than the ribosome (Lindstrom 2009). The regulation of RPs is believed to endow genes with new expression specificity (Xue and Barna 2012). More studies have reported that mutations of RP genes affect the expression of cancer genes, leading to the occurrence of cancers (Zhou et al., 2015). Amsterdam et al. showed that the risk of malignant peripheral schwannoma and other zebrafish cancers increases significantly with mutations in some RP genes (Amsterdam et al., 2004). Human cancers are more likely to develop if mutations occur in the RP genes RPL37, RPS15, and RPS20. Normally, these relevant genes encode RPs that decrease MDM2 E3 ligase activity by binding to MDM2, and they further affect p53 stability and promote cell cycle stagnation, thereby decreasing the risk of cancers (Daftuar et al., 2013).

Ribosomal protein L8 (RPL8) is converted into the large subunit of ribosomes in RP’s L2P family. RPL8 has diverse regulatory functions, including regulating the reproductive cycle of mosquitoes and RNA processing in the intervertebral disc degeneration process (Mazzacano and Fallon, 1996; Yang et al., 2015). Additionally, RPL8 interacts with the transcription factor (TF) Pax6 to change the milk fat traits of dairy cows (Zheng et al., 2019). Emerging evidence suggests that RPL8 has a close relationship with the oncogenesis and progression of tumors. RPL8 was dysregulated in osteosarcoma (Sun et al., 2015) and hepatocellular carcinoma samples (Zhou et al., 2017). RPL8 amplification was closely associated with osteosarcoma pathogenesis (Yang and Zhang, 2013). RPL8 cannot only stimulate T helper (Th) cell cloning but also affect the proliferation of lymphocytes and the expression of cytokines in melanoma patients (Swoboda et al., 2007). Furthermore, in ovarian cancer, protein RPL8 is regarded as a specific tumor antigen (Luo et al., 2002). Although RPL8 is associated with many tumors, the specific biological functions of RPL8 affecting tumor occurrence and development remain unknown.

In an effort to address a glimpse into the mechanisms underlying the effect of RPL8 in tumors, RPL8 (RPL8-OE) level was overexpressed, and global transcriptome sequencing (RNA-seq) experiments were performed. The differences in gene expression and alternative splicing (AS) patterns between the RPL8-OE and control (Ctrl) samples were analyzed to determine whether the dysregulated genes are enriched in tumorigenesis-related pathways and to investigate the pathways through which RPL8 participates in tumor progression. We found that RPL8 may affect the oncogenesis and development of tumors by influencing cell proliferation, adjusting angiogenesis, and regulating inflammatory responses and other related pathways. Furthermore, RPL8 may influence cancer-related DEGs by modulating the splicing patterns of TFs.

## 2. Materials and methods

### 2.1 RPL8 overexpression plasmid design

Primer pairs for the hot fusion cloning were created using the CE Design software (version 1.04). The primer contained a fragment of the *RPL8* sequence and one sequence of the pIRES-hrGFP-1a vector. The forward primer is agcccgggcggatccgaattcATGGGCCGTGTGATCCGT, and the reverse primer is gtcatccttgtagtcctcgagGTTCTCTTTCTCCTGCACAG. The vector was digested by EcoRI and XhoI (NEB) at 37 °C for 2–3 h and then purified by a Qiagen column kit. The plasmid transformation and colony screening were carried out according to the published paper (Tu et al., 2019).

### 2.2 RPL8-OE plasmid transfection into HeLa cells

Experiments were performed using HeLa cells (CCTCC@GDC0009) from CCTCC (China Centre for Type Culture Collection, Wuhan, Hubei, China). RPL8-OE plasmid transfection was performed using Lipofectamine 2000 (Invitrogen, Carlsbad, CA, USA). The HeLa cells were cultured at 37 °C under 5% CO_2_ in Dulbecco’s modified Eagle’s medium with 10% fetal bovine serum (FBS), 100 μg/mL streptomycin, and 100 U/mL penicillin. The cell culture and transfection procedures were performed following the published paper (Tu et al., 2019).

### 2.3 Cell proliferation and apoptosis assays

The MTT assay was used to measure the cell proliferation rate. A total of 5 × 10^3^ HeLa cells/well were inoculated in a 96-well culture plate. After the plates were incubated for 48 h, MTT solution (0.025 mL, 5 mg/mL) was added to each well of the culture plate. HeLa cells were further incubated for 4 h. The plates were centrifuged, and the supernatant in each well was removed. The absorbance of the solution at 490 nm was measured after the colorful formazan crystals produced by MTT were dissolved in DMSO.

Flow cytometry was used to detect apoptosis. The RPL8-OE and Ctrl cells were incubated at 37 °C for 48 h. The living cells were then extracted and washed twice with ice-cold 1 × phosphate-buffered saline. The following procedures were performed according to the previous study (Ma et al., 2020).

Student’s *t*-test was performed to assess the differences between RPL8-OE and the Ctrl groups. The results of each experiment were the mean ± SD. SPSS 19.0 software (IBM Corp., Armonk, NY, USA) was used to analyze the data. *p* < 0.05 was regarded as statistically significant.

### 2.4 RNA-seq library and sequencing experiments

Total RNA was extracted from RPL8-OE and Ctrl HeLa cells using TRIzol (Ambion). The purified RNAs were evaluated for purity and quantity using SmartSpec Plus (BioRad, USA). RNA-seq libraries were constructed using the captured polyadenylated RNAs and the VAHTS Stranded mRNA-seq Library Prep Kit (Vazyme), as previously reported (Li et al., 2018). The RNA-seq libraries were then applied to 150-nucleotide (nt) paired-end sequencing using the Illumina HiSeq X Ten system.

### 2.5 RNA-seq data processing and analysis

Low-quality reads were removed from the raw data by discarding reads with unknown bases (N), adaptors, and low-quality bases (<20) using a FASTX-toolkit (version 0.0.13). Following that, short reads (<16 nt) were also removed. The TopHat2 software (Kim et al., 2013) was used to align the clean reads to the human genome (GRch38), with no more than four mismatches allowed. Reads with the unique genomic location were used to calculate gene expression level as the fragments per kilobase of transcript per million fragments mapped (FPKM) (Trapnell et al., 2010).

### 2.6 Analysis of DEGs and AS

The R Bioconductor package edgeR (Robinson et al., 2010) was used to screen the DEGs between RPL8-OE and Ctrl samples. The false discovery rate (FDR) < 0.05 and fold change (FC) > 2 or < 0.5 were set as the criteria.

The ABLas pipeline was used to calculate the ASEs and RASEs between RPL8-OE and Ctrl samples (Xia et al., 2017). Briefly, based on splice junction sites, 10 ASEs were detected, including exon skipping (ES), cassette exon, alternative 5’ splice site (A5SS) and 3’ splice site (A3SS), intron retention (IR), mutually exclusive exons (MXEs), mutually exclusive 5’ UTRs (5pMXE) and 3’ UTRs (3pMXE), A3SS and A3ES, and A5SS and A5ES. Student’s *t*-test was used to calculate the significance of the ratio alteration of ASEs to identify RASEs by RPL8-OE. ASEs with a *p*-value < 0.05 were considered RASEs.

### 2.7 RT-qPCR experiments for DEGs and RASEs

Reverse transcription and quantitative PCR (RT-qPCR) tests were performed to validate DEGs and RASEs from RNA-seq. For RASEs, isoform-specific primers were designed to amplify the model or alternative forms of the spliced products according to the published method (Liu et al., 2020). Total RNA from the same batch of RNA-seq was prepared in triplicate for each sample. The housekeeping gene GAPDH was used as the internal control. RT-qPCR experiments were performed according to the previous method (Ma et al., 2020). Isoform-specific primers were designed to validate RASEs (Liu et al., 2020). Meanwhile, RT-qPCR experiments were performed to confirm the success of RPL8-OE. The results were compared by unpaired Student’s *t*-test using GraphPad Prism software (GraphPad Software Inc., San Diego, CA, USA).

### 2.8 Functional enrichment analysis

To investigate the enriched functions of DEGs and RASGs, the KOBAS 2.0 server was used to identify the biological process GO terms from the gene ontology (GO) database (Xie et al., 2011). Hypergeometric test was used to calculate the *p*-value and Benjamini-Hochberg FDR was used to control the FDR rate for each pathway.

### 2.9 Motif analysis of the RPL8-regulated DEGs and ASGs

HOMER software (Heinz et al., 2010) was used to calculate the significant motifs (*p*-value < 0.01) in the promoter regions of RPL8-regulated DEGs. TFBSTools (http://www.bioconductor.org/packages/release/bioc/html/TFBSTools.html) package was used to identify the motifs of TFs that contained RASEs by RPL8-OE. TFs were analyzed using JASPAR2020 (http://jaspar.genereg.net/).

## 3. Results

### 3.1 RPL8-OE in HeLa cells significantly promoted apoptosis

**Figure 1:**
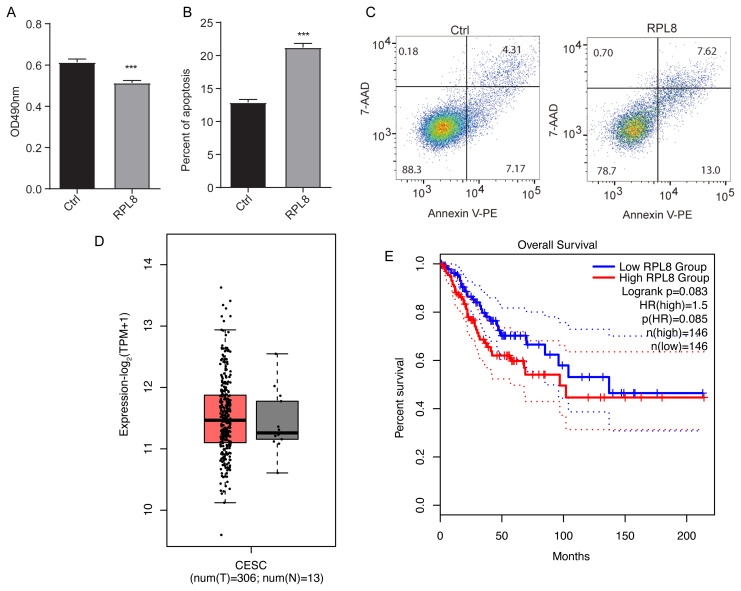
The influence of RPL8 overexpression on proliferation and apoptosis. (A) The cell proliferation in RPL8 overexpression group is measured by the MTT assay. The cell apoptosis in RPL8 overexpression group is measured by flow cytometry. (B) and subsequently by (C) 7-ADD and annexin V assays. ***p < 0.001; 7-AAD, 7-amino actinomycin D; PE, phycoerythrin. (D) Box plot shows the expression pattern of RPL8 from CESC tumor (T, red box) and normal (N, grey box) samples. (E) Line plot shows the overall survival time for CESC patients in both high RPL8 group and low RPL8 group.

To verify the biological effects of RPL8 in HeLa cells, RPL8 genetic vector was transfected and labeled with a green fluorescent protein tag and a flag tag (fused with the target gene) into the cells. The empty vector was transfected into the negative control cells and two RPL8-OE and Ctrl samples were established. After being transfected for 48 h, the cells were collected. The MTT method was used to detect the cell proliferation rate, and the flow cytometry method was used to detect the apoptosis rate. Afterwards, the results between the RPL8-OE and Ctrl groups were compared. We found that RPL8-OE significantly inhibited cell proliferation rate (*p* < 0.001; Figure 1A) and increased the level of cell apoptosis (*p* < 0.001; Figures 1B and 1C). In conclusion, PRL8 inhibits cell proliferation and promotes apoptosis in HeLa cells. For the last step, the expression levels and prognostic values of RPL8 in cervical squamous cell carcinoma and endocervical adenocarcinoma (CESC) were examined from the TCGA database. RPL8 showed a slightly higher expression level in tumor samples (Figure 1D), and the overall survival outcome of CESC patients with higher RPL8 values was worse than that of the patients with lower RPL8 values (Figure 1E).

### 3.2 Analysis of RPL8-regulated transcriptome

**Figure 2:**
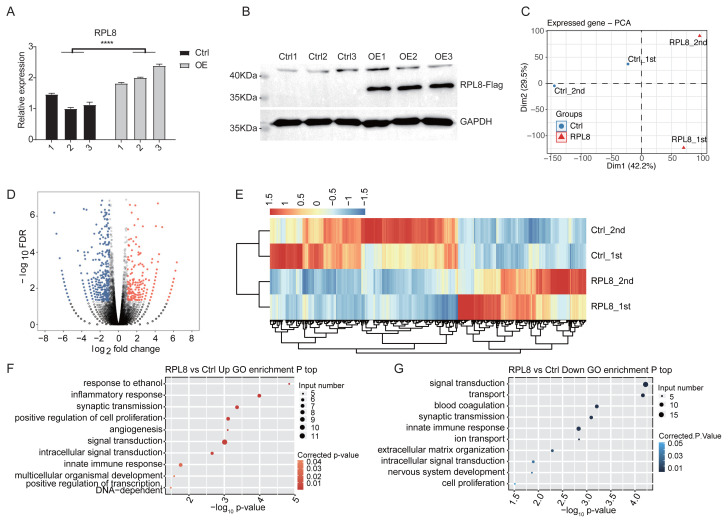
RNA-sequencing analysis of the RPL8-regulated transcriptome. (A) RPL8 expression is quantified using a quantitative reverse transcription PCR assay. Error bars represent mean ± SEM. ***p < 0.001. (B) RPL8 expression is measured by Western blot experiment. (C) Principal component analysis (PCA) of the Ctrl and RPL8-OE groups. (D) The volcano plot displays the identification of RPL8-regulated genes. Upregulated genes are labeled in red and downregulated genes are labeled in blue. (E) Hierarchical clustering of DEGs in the Ctrl and RPL8-OE samples. FPKM values are log2-transformed and then median-centered for each gene. The top 10 GO biological processes of upregulated (F) and downregulated (G) genes.

To decipher how RPL8-OE regulates cell apoptosis, RNAs from RPL8-OE and Ctrl samples were extracted to analyze the global transcriptome profile changes using RNA-seq. First, the RPL8 overexpression levels were confirmed by RT–qPCR experiments. The result showed the increased level of RPL8 in OE samples (*p* < 0.001, Figure 2A). Western blot experiment validated RPL8 overexpression in the protein level (Figure 2B). Two samples from both groups (Ctrl-2, Ctrl-3, OE-2, and OE-3) were selected for RNA-seq. The raw reads were filtered and aligned to the human genome (GRCH38). The detailed cleaned reads and the uniquely aligned reads are presented in Table S1. Afterward, the mRNA length and sequencing depth were normalized for comparing the expression abundance of different sequencing samples and eliminating the deviation between different samples. A total of 28,096 expressed genes were detected from the 2 RPL8-OE samples and the 2 Ctrl samples (Table S2). Principal component analysis (PCA) indicated that the Ctrl samples and the RPL8-OE samples are separated (Figure 2C).

### 3.3 GO analysis of DEGs produced by RPL8-OE

The edgeR package was used to identify RPL8-OE-regulated DEGs (Robinson et al., 2010). The mathematical model of the edgeR package is based on a negative binomial distribution model. Finally, 703 DEGs were identified, comprising 285 up-DEGs and 418 down-DEGs (Table S3). According to FDRs and fold changes, the volcano map showed their distributions (Figure 2D). Hierarchical clustering analysis showed the distinct expression patterns of all the significant DEGs. The heatmap showed a high consistency between the 2 biological replicates and a clear separation between RPL8-OE and Ctrl samples except for several genes (Figure 2E), suggesting that most of these DEGs in HeLa cells were regulated by RPL8-OE.

GO enrichment analyses were performed to further verify the enriched biological processes of DEGs. The top 10 pathways enriched by the up-DEGs were response to ethanol, inflammatory response, synaptic transmission, positive regulation of cell proliferation, angiogenesis, signal transduction, intracellular signal transduction, innate immune response, multicellular organismal development, and positive regulation of DNA-dependent transcription pathways (Figure 2F, Table S4). By contrast, the top 10 functional pathways enriched by the down-DEGs were signal transduction, transport, blood coagulation, synaptic transmission, innate immune response, ion transport, extracellular matrix organization, intracellular signal transduction, nerve system development, and cell proliferation pathways (Figure 2G, Table S4). Among the identified pathways, the inflammatory response, positive regulation of cell proliferation, angiogenesis, and innate immune response pathways are associated with the occurrence and development of tumors.

**Figure 3:**
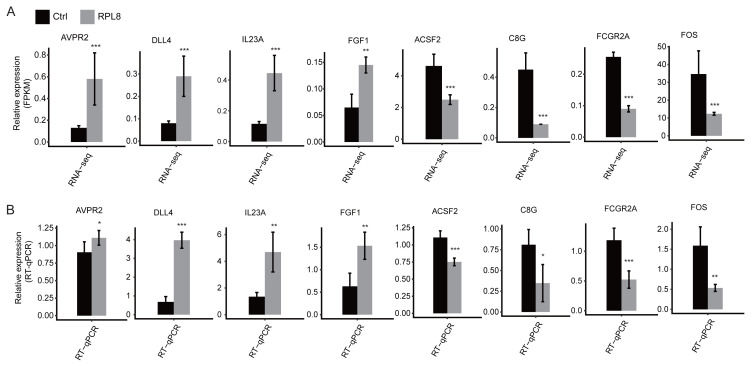
RPL8 regulates gene expression in HeLa cells. (A) Gene expression is quantified using the RNA-sequencing data. FPKM values are calculated using the method explained in Section 2. (B) These genes are validated using qPCR. Error bars represent mean ± SEM. *p < 0.05, **p < 0.01 and ***p < 0.001.

To validate the DEGs and identify their association with cellular dysregulation by RPL8-OE in HeLa cells, RT-qPCR was performed using several randomly selected DEGs from the cancer-related pathways, including *AVPR2*, *DLL4*, *IL23A*, *FGF1*, *ACSF2*, *C8G*, *FCGR2A* and *FOS*. The cancer-related pathways are positive regulation of cell proliferation, angiogenesis, innate immune response pathways in upregulated pathways, and innate immune response pathways in downregulated pathways. We found that *AVPR2*, *DLL4*, *IL23A*, and *FGF1* were upregulated, while *ACSF2*, *C8G*, *FCGR2A*, and *FOS* were downregulated (Figures 3A and 3B). Thus, the RT–qPCR results were consistent with the RNA-seq data, indicating that RPL8-regulated DEGs were credible.

### 3.4 RPL8 regulates the AS pattern in HeLa cells

**Figure 4:**
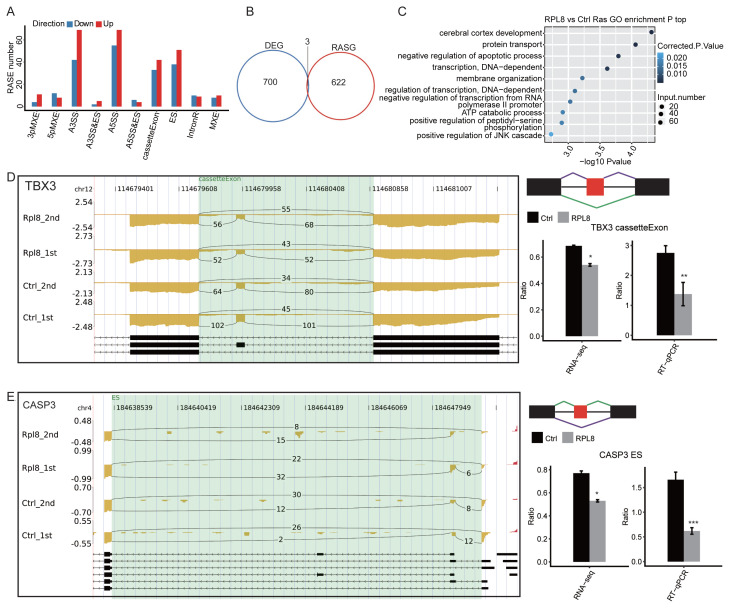
RPL8 regulates the alternative splicing events (ASEs) in HeLa cells. (A) Classification of RPL8-regulated alternatively spliced events. (B) Overlap analysis of RPL8-regulated DEGs and alternatively spliced genes (ASGs). (C) The top 10 enriched GO biological processes of PL8-regulated ASGs (RASGs). (D) RPL8 regulates the alternative splicing of *TBX3*. The IGV-Sashimi plot in the right-top panel shows a cassette exon event. The right-bottom panel displays the altered ratios of ASEs in RNA sequencing and in quantitative reverse transcription PCR (RT–qPCR) assay. (E) RPL8 regulates the alternative splicing of *CASP3*. The IGV-Sashimi plot in the right-top panel shows a cassette exon event. The right-bottom panel displays the altered ratios of ASEs in the RNA sequencing and RT-qPCR assays. The left panel presents the reads distribution of each ASE, with the transcripts of each gene shown below. The schematic diagrams depict the structures of ASEs, AS1 (purple line), and AS2 (green line). Constitutive exon sequences are denoted as black boxes, intron sequences as the horizontal line (right panel, top), alternative exons as red boxes, and introns as purple boxes. The right-bottom panel shows RNA-seq quantification and RT-qPCR validation of ASEs. Error bars represent mean ± SEM. * *p* < 0.05, **p < 0.01, and ***p < 0.001.

RPs could regulate the AS of mRNAs, including RPS26 (Ivanov et al., 2005) and RPL10a (Takei et al., 2016), which inspired us to explore the AS changes caused by RPL8-OE. By aligning the RNA-seq data to human reference genome sequences, we extracted uniquely mapped reads and matched them to the annotated exons. After that, the spliced reads were aligned to multiple exons to perform the AS analysis. A total of 248,257 exons, accounting for 67.58% of the 367,321 annotated exons in the reference genome, were selected. A total of 358,131 splice sites were detected using TopHat2 (Kim et al., 2013) and classified into 193,851 new splice sites and 164,280 known splice sites. Finally, a total of 20,663 annotated alternative splicing events (ASEs), accounting for 25.42% of the total genome, were detected using ABLas. Student’s *t*-test was used to analyze the differences in ASEs between RPL8-OE and Ctrl groups. A *p*-value < 0.05 was set as the criteria to identify RPL8-regulated ASEs (RASEs). RASEs with T-value > 0 were recorded as up RASEs, which implied that the proportion of a splicing type in RPL8-OE samples is higher than that of the control samples, whereas RASEs with T-value < 0 were recorded as down RASEs, which implied that the proportion of a splicing type in RPL8-OE samples is lower than that of the Ctrl samples. By comparing the RPL8-OE and the Ctrl groups, we found 370 upregulated RASEs and 302 downregulated RASEs. These RASEs included 15 3pMXE, 20 5pMXE, 111 A3SS, 124 A5SS, and 89 ES (Figure 4A). To examine whether the genes with RASEs were also under the transcriptional regulation of DEGs, an integrated analysis of RASE genes (RASGs) and DEGs was performed. Three genes were extracted and showed significant differences both at the expression and AS levels (Figure 4B), suggesting that most of the RASGs were independent of their expression level changes.

**Figure 5:**
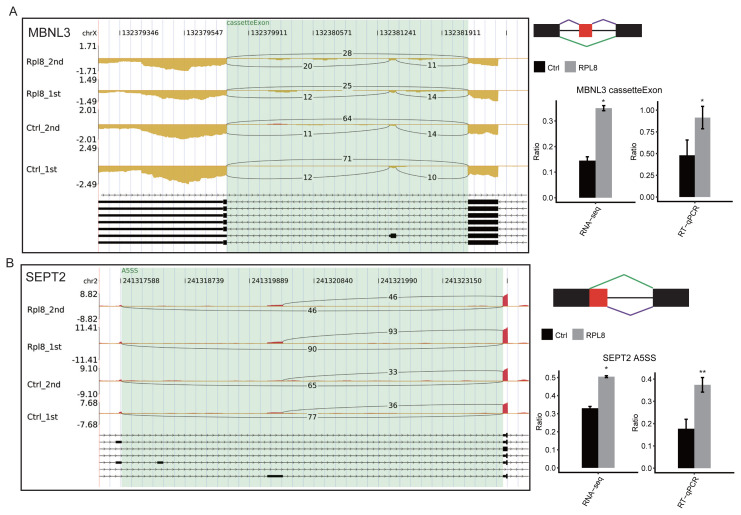
The same as described in Figures 4D–4E, but for the cassette exon of MBNL3 (A) and alternative 5’ splice sites of SEPT2 (B).

Moreover, a GO functional enrichment analysis of the RASGs was performed to explore their enriched pathways. With GO annotation of the whole gene set as background, the GO terms were obtained and displayed the top 10 terms. The GO enrichment analysis showed that the terms included cerebral cortex development, protein transport, negative regulation of the apoptotic process, transcription-related pathways, and membrane organization (Figure 4C, Table S4). In total, 25 genes were implicated in the negative regulation of apoptosis, including *CASP3*, *VHL*, *MYD88*, *RAF1*, *CAMK1D*, and *UBA52*. These findings indicate that RPL8 could regulate the AS of tumor-related genes. We then used RT–qPCR assay to validate the RASEs by RPL8-OE. Several RASEs and associated genes were randomly selected, including *TBX3* (cassette exon), *CASP3 (*ES), *MBNL3* (cassette exon), and *SEPT2* (A5SS). An IGV-sashimi plot showed cassette exon or exon skipping events (Figures 4D, 4E, and 5A) and alternative 5’ splice sites (Figure 5B). The RT-qPCR results of the 4 RASEs showed high consistency with the RNA-seq results. Taken together, these results showed that RPL8 could regulate the pattern of ASEs in HeLa cells.

### 3.5 TFs from RASGs by RPL8-OE were tightly associated with DEGs

**Figure 6:**
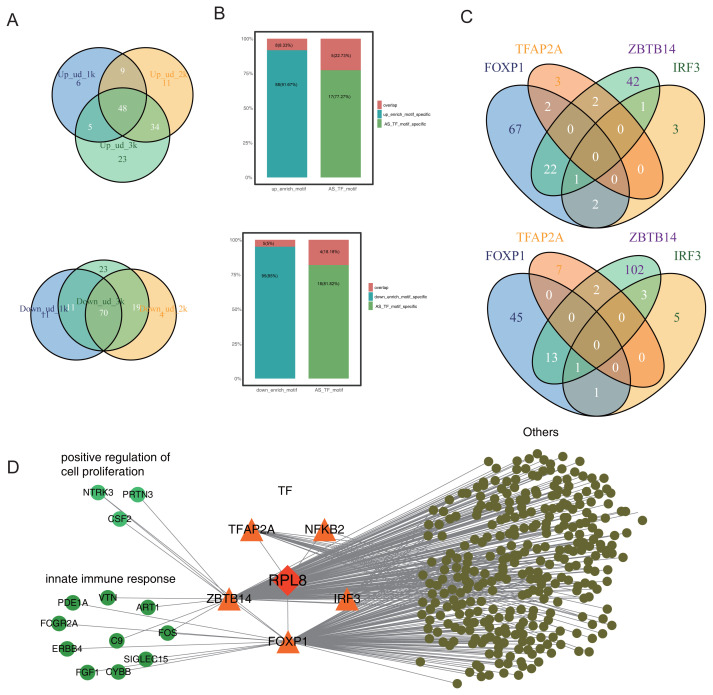
Analysis of RPL8-regulated and DEG-associated alternative splicing genes. (A) The Venn diagram shows the overlapping motifs between 1K and 3K away from the transcription initial site. (B) Overlapping motifs of RPL8-regulated AS-TFs and DEGs (upregulated DEGs in the top panel and downregulated DEGs in the bottom panel). (C) The overlapping motifs of the 4 top AS-TFs (FOXP1, TFAP2A, ZBTB14, IRF3) in up- (top panel) and downregulated (bottom panel) DEGs. (D) The network between RPL8-regulated AS-TFs and targeted DEGs involved in cell proliferation and immune response pathways.

In addition to the cancer-related pathways identified from the GO analysis of RASGs, some pathways were found to be associated with DNA-dependent transcription and included transcription factors (TFs). Thus, we hypothesize that RPL8 may influence the expression of DEGs by regulating the AS patterns of TFs. To validate this hypothesis, RASGs were subjected to TFBSTools to identify TFs. Afterward, HOMER software (Heinz et al., 2010) was employed to calculate the enriched motifs in the promoter regions (1K, 2K, and 3K away from the transcription start sites) of all DEGs. The results showed that there were 48 common motifs in the up-DEGs and 70 common motifs in the down-DEGs (Figure 6A). The enriched motifs that were identified at least in two groups of the promoter sets were selected and 96 motifs in up-DEGs and 100 motifs in down-DEGs were obtained. TFs were verified using the JASPAR2020 database. Then, TFBSTools package was used to correspond to these common motifs of the DEGs and AS-regulated TFs (AS-TFs). Eight out of these enriched motifs in up-DEGs overlapped with 5 motifs of RPL8-regulated AS-TFs, while 5 motifs in down-DEGs overlapped with 4 motifs of RPL8-regulated AS-TFs (Figure 6B). The AS-TFs (*FOXP1*, *TFAP2A*, *ZBTB14*, and *IRF3*) contain the most enriched motifs. The Venn diagram showed the overlapping genes containing motifs of these 4 TFs (Figure 6C). The network between RPL8-regulated AS-TFs and the targeted DEGs is presented in Figure 6D, demonstrating that RPL8 may regulate the expression of genes involved in the positive regulation of cell proliferation and innate immune response by modulating the AS of *ZBTB14* and *FOXP1* (Figure 6D). These results indicate that PRL8 may regulate the AS of TFs, which may influence the DEGs.

## 4. Discussion

RPs participate in numerous biological regulatory mechanisms ranging from the regulation of protein synthesis to cell death induction and cell cycle arrest through inhibiting the protein ubiquitination pathways, thereby affecting the oncogenesis and development of tumors (Amsterdam et al., 2004; Goudarzi and Lindstrom, 2016; Ebright et al., 2020). RPs RPL37 and RPS15 could regulate the Mdm2-p53-MdmX network, and RPS15 is known to modulate tumor occurrence and development (Daftuar et al., 2013). RPL15 overexpression could influence the cell proliferation and metastasis of gastric cancer (Wang et al., 2006) and breast cancer (Ebright et al., 2020). RPL8, a constitutive RP gene, is implicated in multiple tumor types, including liver cancer, melanoma, and ovarian cancer (Swoboda et al., 2007; Zhou et al., 2017), while the mechanisms of RPL8 affecting related biological pathways in tumors are still unclear. In this study, RPL8 was overexpressed in HeLa cells to investigate its biological functions and regulatory profiles. The results showed that the RPL8-regulated gene network is not limited to p53-related pathways and that RPL8 can regulate the expression and AS of cancer-related genes. RPL8 can promote cellular apoptosis and regulate gene expression, perhaps by modulating the AS of the associated TF genes.

We found that RPL8 was able to regulate the expression levels of 703 genes. It remains unclear that how RPL8 regulates the transcriptional levels of genes. Interestingly, a recent study reported that RBPs influence gene transcription by directly interacting with the hotspots in the genome, particularly gene promoters (Xiao et al., 2019). RPL8 may regulate gene transcription through this mechanism. By analyzing the functions of DEGs, we found that the DEGs by RPL8-OE were enriched in cancer-associated pathways, including inflammation, positive regulation of cell proliferation, angiogenesis, and innate immune response. Among these DEGs, *AVPR2*, *DLL4*, *IL23A*, and *FGF1* were upregulated; *ACSF2*, *C8G*, *FCGR2A*, and *FOS* were downregulated. The literature retrieval results show that these genes are associated with oncogenesis and tumor progression. AVPR2 plays a potential role in the development of canine mammary tumors. In addition, as a selective vasopressin analog, AVPR2 may be served as a therapeutic target (Benavente et al., 2020). IL-23 was reported to promote tumor incidence and growth (Langowski et al., 2006). DLL4 inhibition decelerates tumor growth by deregulating angiogenesis (Ridgway et al., 2006). FGF1 could facilitate neoplastic progression, thus playing an important role in human salivary glands and breast cancer (Mazzacano and Fallon, 1996; Yoshimura et al., 1998). ACSF2 may participate in metastatic melanoma by regulating ferroptosis (Gagliardi et al., 2020). FCGR2A polymorphisms are correlated with metastatic colorectal cancer (Kjersem et al., 2014). FOS affects tumor progression by regulating the Ras-ERK or PI-3K-AKT pathway (Tice et al., 2002). Meanwhile, several studies showed that RPL13 and RPS19 extensively participated in immune and inflammatory responses in many diseases (Filip et al., 2009; Poddar et al., 2013; Guan et al., 2021). The results indicate that RPL8 could participate in the immune/inflammatory responses in cancer cells by regulating the expression levels of cancer-associated genes. Meanwhile, this study has some limitations, including the small sample size, the heterogeneity of DEGs, the only one cell line in experiments, the polyadenylated RNA enrichment method for RNA-seq but not rRNA depletion, and the lack of molecular mechanisms of RPL8 regulatory functions. Further studies are necessary to fill these gaps and to generate more solid datasets to reach a firm conclusion.

In addition to regulating DEGs, RPL8 also regulates the AS of hundreds of genes. RPs could regulate the AS of mRNAs. RPS26 could interact with its pre-mRNAs to regulate its own AS (Ivanov et al., 2005). RPL10a specifically binds to the fragment between the 2 A5SSs in its pre-mRNAs to affect the choice of splice sites (Takei et al., 2016). We found that RPL8 was able to regulate the AS of other genes and that many RASGs were enriched in tumor pathways. For example, RASGs were involved in renal cancer, colorectal cancer, and virus-associated cancer. Genes *TBX3*, *CASP3*, *MBNL3*, and *SEPT2* were enriched in tumor pathways and their AS level changes were confirmed by RT-qPCR experiments. *TBX3* promotes melanoma formation and invasion (Peres and Prince, 2013). Activation of *CASP3* transcription has been found to suppress the proliferation of cancer cells and to promote cell apoptosis in multiple cancers (Li et al., 2016; Luo et al., 2018; Zhu et al., 2019). *MBNL3* participates in paclitaxel resistance in ovarian cancer (Sun et al., 2021). *SEPT2* is required for the progression of human breast cancer via MEK/ERK activation (Zhang et al., 2016). RASGs were also enriched in negative regulation of apoptosis, consistent with the phenomenon that RPL8-OE increased the apoptotic levels of HeLa cells. This result suggests that RPL8 can regulate the progression of cancer cells by affecting their AS levels. Therefore, RPL8 regulates cancer-related ASGs in HeLa cells.

Furthermore, RPL8-regulated RASGs were enriched in DNA-dependent transcription-related pathways. We hypothesize that RPL8 may influence DEGs by regulating AS-TFs. There were 8 and 5 overlapping motifs from up- and downregulated DEGs, respectively. AS-TFs FOXP1, TFAP2A, ZBTB14, and IRF3 contain the largest number of motifs and are most likely to regulate the expression levels of corresponding DEGs. We also found that the potential DEG targets of AS-TFs were enriched in the innate immune response and positive regulation of cell proliferation, suggesting that RPL8 may influence cancer-related DEGs by regulating the AS of these TFs. Meanwhile, this indirect link between AS-TFs and DEGs is only based on the bioinformatics analysis and needs to be further validated by additional experiments.

In summary, this study provides profound insights into the possible functions of RPL8 in the development of cancers. We found that RPL8 can regulate transcriptome profiles, including gene expression and AS, which is highly associated with the pathogenesis and progression of cancers. We also propose a regulatory model between DEGs and RASGs by analyzing TFs with splicing changes. This study reveals the important roles of RPL8 in regulating cancer cells at transcriptional and posttranscriptional levels, highlighting its potential value as a therapeutic target in cancer treatment.

## Supplementary Information

Table S1 Summary of sample names, description, the RNA-seq sequencing information and mapping results in each sample.

Table S2 Gene expression level (FPKM).

Table S3 Differential expression of genes between RPL8-OE and control.

Table S4 Enrichment analysis of functional GO BP terms.

## Data Availability

RNA-sequencing data in the present study have been deposited in NCBI’s Gene Expression Omnibus and are accessible through GEO (https://www.ncbi.nlm.nih.gov/geo/query/acc.cgi?acc=GSE161696).

## Appendix

3pMXE: mutually exclusive 3’UTRs
5pMXE: mutually exclusive 5’UTRs
A3SS: alternative 3’ splice site
A5SS: alternative 5’ splice site
ASEs: alternative splicing events
ASGs: alternatively spliced genes
CC: cervical carcinoma
DEGs: differentially expressed genes
EMT: epithelial-mesenchymal transition
ES: exon skipping
FC: fold change
FDR: false discovery rate
FPKM: fragments per kilobase of transcript per million fragments mapped
GAPDH: glyceraldehyde-3-phosphate dehydrogenase
GO: gene ontology
MMP-9: matrix metalloproteinase 9
MXEs: mutually exclusive exons
RASEs: regulated alternative splicing events
RPs: ribosomal proteins
UDG: uracil-DNA glycosylase
VEGF: vascular endothelial growth factor
